# Longitudinal Patterns of Sexual Recidivism by Age Over a 25-Year Follow-Up in California

**DOI:** 10.5964/sotrap.3667

**Published:** 2022-04-19

**Authors:** Stephanie Brooks Holliday, Shoba Sreenivasan, Sarah Cusworth Walker, Victor Jordan, Allen Azizian, Thomas Garrick

**Affiliations:** 1 RAND Corporation, Santa Monica, CA, USA; 2 USC Keck School of Medicine, Los Angeles, CA, USA; 3 California Department of State Hospitals, Sacramento, CA, USA; 4 University of Washington, Seattle, WA, USA; 5 California Department of Corrections and Rehabilitation, Sacramento, CA, USA; 6 California State University, Fresno, Fresno, CA, USA; Centre for Criminology (Kriminologische Zentralstelle – KrimZ), Wiesbaden, Germany

**Keywords:** age, sexual offending, recidivism, longitudinal

## Abstract

This study followed 146 sexual offenders released from prison custody for a period of 25-years. Overall, 34% of individuals committed at least one sexual reoffense in the 25 years following release from incarceration. Most sexual recidivism occured within the first 15-years following release. The highest rates of sexual recidivism were observed for individuals under 34 years at release from incarceration, for whom recidivism steadily increased over time before peaking at 42% at 25 years. The mean age at reoffense was 42.51. Age was significantly associated with sexual recidivism at 5 years, but not at subsequent follow-up periods. These findings suggest that long-term patterns of sexual recidivism may be related to age at release. It will be important for future research to explore the characteristics of individuals who commit sexual offenses that may contribute to reoffending risk, and examine the effectiveness of policies and practices designed to mitigate recidivism.

Risk factors such as history of previous sexual offending; markers of sexual deviance, such as having male victims or stranger victims; and factors reflecting general criminality, such as history of non-sexual justice system involvement or juvenile delinquency all contribute to sexual recidivism risk (Hanson & Bussière, 1998). There is evidence that the likelihood of sexual recidivism appears to decline after the first five years post-release (Hanson, Harris, Helmus, & Thornton, 2014; Hanson, Harris, Letourneau, Helmus, & Thornton, 2018). An analysis based on 7,740 sexual offenders pooled across 21 studies found that 22% of high-risk individuals reoffended in the first five years after release. However, if high-risk individuals were able to avoid reoffending in the first ten years after release, the rate of recidivism in the next five years was only 4.2% (Hanson et al., 2014). In a follow-up study, Hanson and colleagues (2018) found that risk of sexual recidivism declined with time across risk groups. As they stated, “most individuals eventually resembled individuals with no prior history of sexual crime” (p. 55). Together, these studies highlight that risk of sexual recidivism declines appreciably the longer individuals remain in the community offense free. Desistance appears to be the norm for sexual recidivism, even for those who are deemed at initial evaluation to be high-risk (Hanson, 2018).

Age may be an important contributing factor to the declining rate of sexual recidivism over time. Over twenty years ago, Hanson and Bussière (1998) published their seminal meta-analysis examining predictors of sexual recidivism using data from 21 follow-up studies with a collective sample of 6,969 individuals. This meta-analysis demonstrated a small but consistently negative relationship between age and sexual recidivism: the older the individual who committed a sexual offense, the smaller their risk for sexual reoffending. Several years later, Hanson (2002) conducted another large-scale examination of 4,673 individuals with sexual offenses from ten samples. This study also identified a decrease in sexual recidivism with age at release. The effect was not uniformly linear, with the pattern of recidivism over time varying by type of offense. For example, individuals who committed extra-familial child molestation tended to remain at higher levels of risk in their 20s and 30s, plateauing in their 40s with a drop-off occurring after age 50; by contrast, risk among those who commited rape or incest reduced in a more linear fashion. However, although there were differences across offense type, sexual recidivism risk generally tended to decrease with age at release. This is consistent with other research finding lower risk of sexual recidivism among individuals who were older at age of release – and especially for those who were over age 60 at release, with very low (or no) sexual recidivism occurring regardless of the nature of previous sexual offending (Barbaree et al., 2003; Hanson, 2006).

To explore the role of age in more detail, Hanson (2006) used a combined cross-sectional sample of 3,425 sex offenders across eight studies to create four age bands using age at release: those 18-30, those in their 40s, 50s, and over 60 using a five-year follow-up. This study found a curvilinear effect of age, with those released in their 30s demonstrating the greatest risk for sexual reoffense (charges or convictions), a steady decline into the 40s and with very few instances of sexual re-offending among those released at age 60 or over (2.4%).

However, there are limitations to existing research on the role of age in desistance from sexual offending. First, even longer-term recidivism studies tend to report only 10 year follow-up periods (e.g., Nicholaichuk, Olver, Gu, & Wong, 2014). There is also greater need to consider the interplay between aging and time individuals spend offense-free in the community over long-term follow-up periods. For example, the previously described study by Hanson and colleagues (2014) explored sexual recidivism rates for three age groups: 18-30 years at release, 30-50 years at release, and 50+ years at release. They found younger individuals had the lowest recidivism rate in the first five years after release; however, relative rates of recidivism were similar across age groups from years 6-10 and years 11-15. This demonstrates that, across age groups, likelihood of reoffending declines with time, and raises questions about the contributions of age and time offense-free in the community in desistance from sexual offending.

The present study leverages a unique dataset collected from the California Department of Corrections and Rehabilitation (CDCR), which included 25-year recidivism data within a sample of individuals who committed sexual offenses and were released from prison custody. Our goal was twofold: (a) to describe patterns of sexual recidivism across the 25 year follow-up period, and (b) to explore how rates of sexual recidivism varied by age at release.

## Method

### Procedures and Participants

The project was reviewed and approved by the CDCR Research Board. This was a convenience sample consisting of a subset of a total of 5,898 individuals with a history of sexual offenses identified by CDCR as having been released from prison in the sampling period of interest (January 1, 1989 to December 31, 1990). The cases included individuals who had committed a sexual offense sometime in their criminal histories (prior to or including the commitment period before first release in 1989/1990) and were required to register as a sex offender. The sample consisted of the first 177 case files that were available for review during the period of the study (January 1, 2007 – December 1, 2008) from the total sample of 5,898. The method of file selection was based on staff availability to pull specific files from storage (at headquarters) and send the boxes to the region where the reviewers were located. Although this number fell short of even the minimum targeted 10% of the sample the researchers had planned, the primary restriction on sample accessibility was the prison system’s limited staff resources and regulations that restricted pulling of files to records in the narrow time frame allocated for this task. Of these files, complete data on the variables of interest were abstracted from 146 files. Within the sample of 146 participants, most offenders were Black (42.5%, *n* = 62), followed by White (29.5%, *n* = 43), Hispanic (17.8%, *n* = 26), and other (5.5%*, n* = 8) (information was missing for *n* = 7 individuals). The mean age at release was 37.90 (*SD* = 7.08; range = 21-60). Regarding sexual offending history, 69.9% (*n* = 102) had a history of crimes against adults, 11.0% (*n* = 16) had a history of crimes against children, and 19.2% (*n* = 28) had a history of both types of offenses.

### Measures

#### Sexual Recidivism

Sexual recidivism was broadly defined to include either charges or convictions for criminal sexual behavior during the 25-year follow-up period after the first release date (in 1989/1990) and as noted within the California Department of Justice criminal history Record of Arrest and Prosecutions (RAP) sheet (generated 09/2014). This included sexual offenses involving force and violence or substantial sexual conduct, such attempted offense of rape with force; rape with threat of future retaliation; rape or penetration of genital or anal openings by foreign objects; rape in concert by force or violence; spousal rape with threat of future retaliation; non-consensual sodomy; non-consensual oral copulation; and all penal code sections of lewd acts on a child under 14, 16, or 18. Noncontact offenses such as exhibitionism, voyeurism, or annoying/molesting a child were also included as sexual offenses. Arrests or convictions for solicitation/prostitution and pimping were not classified as sexual offenses.

### Statistical Analysis

Analyses were largely descriptive in nature, given the small sample size and lack of information regarding long term trajectories of sexual offending in the literature. To explore patterns of sexual recidivism and age across the 25-year follow-up period, we initially classified individuals into five-year age bands reflecting the individuals’ age at the time of their release from incarceration for the index sexual offense. Given the small number of individuals in the youngest age groups and the oldest age groups, we then collapsed these age groups into four age categories: < 35 years, 35-39 years, 40-44 years, and 45-64 years. We then descriptively examined rates of new sexual recidivism during each five-year follow-up period through 25 years. Finally, we conducted a series of logistic regression analyses to determine if age was associated with sexual recidivism at each follow-up period.

## Results

**Figure 1:**
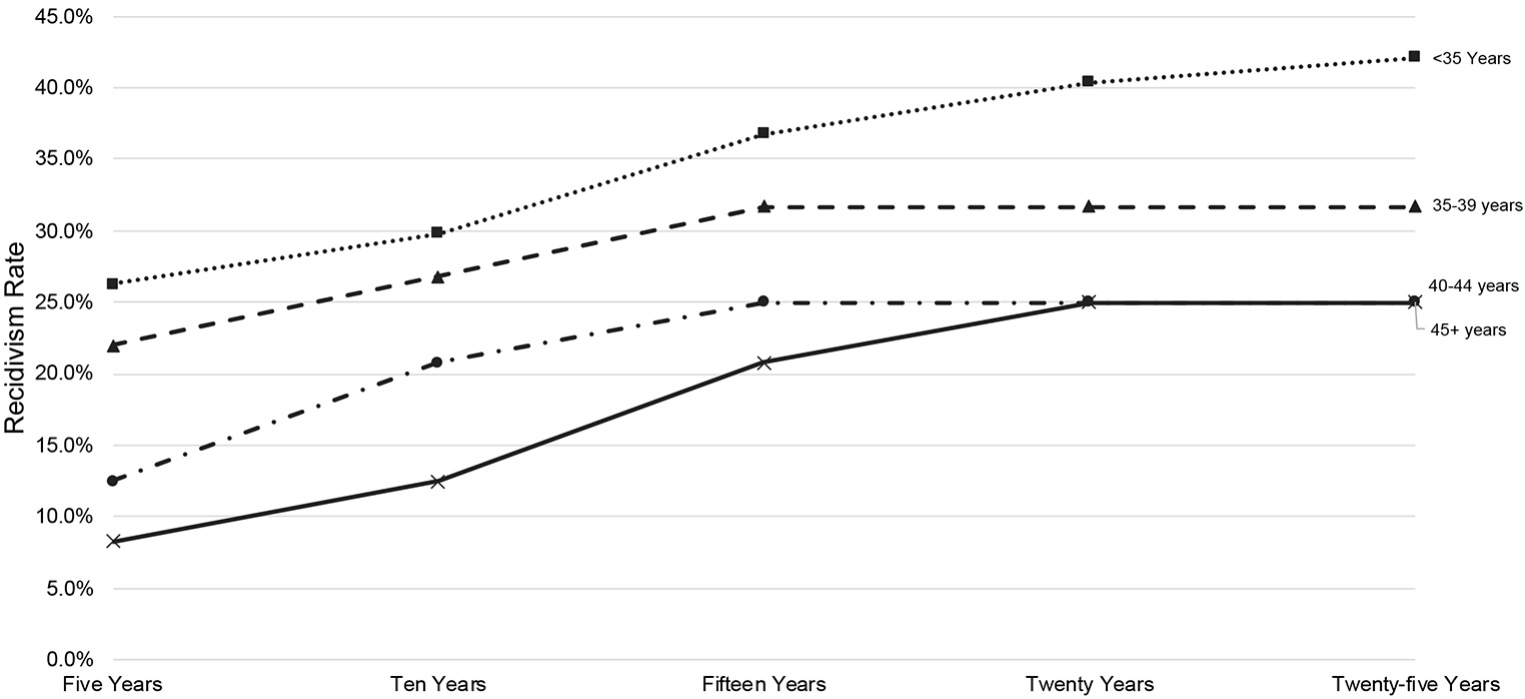
Cumulative Sexual Recidivism by Age Band

Table 1 presents the rates of sexual recidivism at each follow-up time period. Overall, 34% of individuals committed at least one reoffense in the 25 years following release from incarceration. Rates of reoffending across time and by age group are also presented in Table 1. The highest rates of sexual recidivism were observed for the < 35 year age band, for whom sexual recidivism steadily increased over time before peaking at 42.1% at 25 years. Figure 1 displays sexual recidivism over time for each age band. This figure displays cumulative sexual recidivism at each time point; that is, anyone who recidivated at 5 years is also represented in the 10 year sexual recidivism band. Therefore, any increases reflect new sexual recidivism events. For most age bands, sexual recidivism rates plateaued at around 15 years (i.e., there were few new sexual recidivism events after this time) (see Figure 1). Among the 49 individuals who reoffended, the mean age at reoffense was 42.51 (*SD* = 8.24, range = 29 to 65).

**Table 1:** Cumulative Sexual Recidivism, Overall and in Each Age Group, by Follow-Up Period

Follow-Up Period	Overall		< 35 Years		35-39 Years		40-44 Years		45-64 Years	
	(*n* = 146)		(*n* = 57)		(*n* = 41)		(*n* = 24)		(*n* = 24)	
	*n*	%	*n*	%	*n*	%	*n*	%	*n*	%
Recidivated at 5 Years	29	19.9	15	26.3	9	22.0	3	12.5	2	8.3
Recidivated at 10 Years	36	24.7	17	29.8	11	26.8	5	20.8	3	12.5
Recidivated at 15 Years	45	30.8	21	36.8	13	31.7	6	25.0	5	20.8
Recidivated at 20 Years	48	32.9	23	40.4	13	31.7	6	25.0	6	25.0
Recidivated at 25 Years	49	33.6	24	42.1	13	31.7	6	25.0	6	25.0

Logistic regression analyses indicated that age at release (using a continuous version of the variable) was a significant predictor of sexual recidivism at 5 years. Individuals who were older at release were less likely to reoffend, *OR* = 0.929, 95% CI [0.865, 0.997], *p* = .041. Age at release was also marginally associated with sexual recidivism at 10 years, *OR* = 0.943, 95% CI [0.886, 1.003], *p* = .064. However, there was no significant association between age at release and sexual recidivism at 15 years, *OR* = 0.960, 95% CI [0.909, 1.013], *p* = .136, 20 years, *OR* = 0.959, 95% CI [0.909, 1.011], *p* = .120, or 25 year follow-up, *OR* = 0.956, 95% CI [0.907, 1.009], *p* = .101).

## Discussion

We explored long-term sexual recidivism by age group over a 25 year period following release from incarceration. Overall, 34% of individuals committed at least one reoffense in the 25 years following release from incarceration, which is a substantial base rate of sexual recidivism. Our results suggested that most sexual recidivism occurs within the first 15 years following release from incarceration. Interestingly, this was observed across most age bands, including those who were 45 years old or older at release, meaning that these individuals were still recidivating even into their 60s. Previous work has found very low recidivism rates among individuals released in their 60s and 70s, and it has been hypothesized that factors such as declining libido account for this (e.g., Barbaree et al., 2003). The fact that ongoing sexual recidivism was observed among older individuals in this sample highlights that increased time in the community – and therefore additional opportunity to reoffend – may also influence the likelihood of sexual recidivism. That said, prior work has suggested that the longer individuals remain offense-free in the community, the lower their ongoing risk of sexual recidivism (Hanson et al., 2014). This may also be supported by our finding that age at release was only predictive of recividism at 5 years post-release. Together, these findings underscore the importance of further exploring the influence of age at release versus time in the community in influencing recidivism.

Other research provides some insight into the factors that may contribute with longer-term sexual recidivism by certain age groups. The nature of sexual offenses may be one such factor. For example, Prentky and Lee (2007) examined the effect of age-at-release on sexual recidivism with a 25-year follow-up of a single cohort of individuals committed to a state psychiatric hospital as “sexually dangerous.” This sample included 136 individuals who had committed rape and 115 individuals with charges against children. Among individuals who committed rape offenses, risk of reoffending was approximately 28% for five years post-release and then declined. For those with charges against children, sexual recidivism was observed up to 21 years post-release, peaking in their 30s (42%) and plateauing in their 40s (36%), with a drop evident only after age 50 (23%) and 60s (17%). That said, this study had a unique population – individuals who were civilly committed between 1959 and 1985 due to their sexual dangerousness – which may have been a higher risk population than those released from prison. This may account for the much higher rates of sexual recidivism among individuals in their 50s and 60s than found in other studies (Barbaree et al., 2003; Hanson, 2006). Given our small sample size and the fact that 19% had a mixed offending history, it was not possible for us to further explore the influence of offense type, but this is an important area for ongoing research.

As alluded to previously, it is generally argued that lower rates of sexual recidivism in older populations of individuals who have committed sexual offenses is a function of both risk and physiological factors (Helmus, Thornton, et al., 2012). That is, individuals who are older may have less of a long-standing track record of antisocial behavior, but also experience physiological changes such as reduced sex drive. Along these lines, it may be that age at release is not the best predictor of sexual recidivism, but rather that age at offense is a better indicator of ongoing recidivism risk (Rice & Harris, 2014). Evidence is mixed as to whether age at release is predictive of sexual recidivism (Rice & Harris, 2014; Thornton, 2006). The predictive value of age at release may also depend on the length of incarceration; that is, for someone who serves a fairly short sentence, the distinction between age at offense and age at release may be negligible. But someone who serves a longer sentence may be much younger at the time of offense than the time of release. For these individuals, the question is whether earlier onset of offending – a marker of an antisocial pattern – will have a stronger impact on likelihood of reoffending than age-related factors at release (e.g., physiological changes). It will be important for future research to explore the factors that may explain these different patterns of sexual recidivism by age band, exploring the combined influence of both age at offense and release.

### Limitations

There are limitations of this study. In particular, very few individuals in the sample were released in certain age groups, especially those less than 30 years old (*N* = 7) or greater than 50 years old (*N* = 11). Therefore, we were unable to explore more granular patterns of sexual recidivism for those age groups, making it difficult to draw firm conclusions about the role of age at release in dictating patterns of sexual recidivism over time for those extremes. Another limitation is that, as described above, our sample included individuals who had committed a sexual offense prior to *or* including the commitment period before first release in 1989/1990 and were required to register as a sex offender. Although in some instances the post-release follow-up could conceivably be longer than 25-years with this method, the archival identification of individuals with sexual offenses released from custody was based on sex offender registration status. As such, it was not possible to restrict inclusion to only those who were currently serving a prison term for a sexual offense. Moreover, this broader identification method provides potential clinical utility for forensic civil commitment assessments, such as for Sexually Violent Predator (Weinberger, Sreenivasan, Azizian, & Garrick, 2018) that target all pending parolees with current or past adjudications for sexual offenses.

It was also difficult for us to account for opportunity to offend post-release. That is, some individuals may have been re-arrested or re-convicted on a different charge. Although we had information regarding sentences following subsequent crimes (both non-sex and sex offenses) for some individuals in this sample, these data were missing for others, and it is unclear if it was missing at random. Also, for some individuals, we had information indicating that they were sentenced, but without a specific length on the sentence. We also know that some proportion of participants were on community supervision following release, which may also affect likelihood of re-offense. Relatedly, we did not have data on individuals who may have died over the follow-up period. Mortality likely varied across the different age bands – especially affecting those in older groups, such as individuals who were over 55 at age of release. Because individuals who died had less opportunity to reoffend, especially as they aged, this is an important limitation that should be taken into consideration when interpreting results. Finally, this small sample should be considered a sample of convenience given that the researchers did not have the ability to randomly select cases from the total sample pool. Recividism rates in our study were also higher than those found in recent studies from California (Lee, Hanson, Fullmer, et al., 2018) and may reflect the limitations of our sample of convenience; that is, the risk for oversampling recidivists whose files may be easier to access due to “revolving door” releases and incarceration. Recidivism rates are also influenced by how sexual cases are adjudicated; that is, more recently, sentences for sexual offenses have been longer and particularly so for repeat sexual offenders and may reflect low observed base rates of sexual recidivism in recent California samples (California Department of Corrections & Rehabilitation, 2018).

### Strengths and Future Directions

There are also certain strengths of this study. In particular, we had data on a longitudinal cohort over a substantial outcome period of 25 years. Our understanding of sexual recidivism over this long-term time frame is still limited, and this study makes an important contribution in this way. This study also forms the foundation for future research related to age and longer-term sexual recidivism. For example, though large studies have explored patterns of persistence versus desistance of sexual offending and how this varies by age at release from incarceration, there are still few studies reporting such long-term follow-up periods as in this study.

Ultimately, understanding these patterns has important implications for risk assessment and risk management efforts. For example, in the previously described study by Hanson and colleagues (2018), sexual recidivism markedly declined as the follow-up was extended. Though the cumulative recidivism rate increased (from 9.1% at 5 years to 18.5% at 20 years), it did so with very few additional offenders: at 20 years, only one new sexual recidivist was added. Declines in sexual recidivism were evident even when individuals were categorized by risk level. They concluded that at 10 to 15 years post-release, the likelihood of committing a new sexual crime was extremely low – equivalent to the likelihood of an individual with a history of non-sexual offending committing a sexual offense, except for those in the highest risk group. It may be that a combination of factors such as aging and release supports, such as housing and employment, may all play a part in the reduction of sexual recidivism – but exploring the actual impact of such policies and practices is an important next step.
